# Relation of body surface area-to-mass ratio to risk of exertional heat stroke in healthy men and women

**DOI:** 10.1152/japplphysiol.00597.2023

**Published:** 2024-01-18

**Authors:** Kathryn M. Taylor, Gabrielle E. W. Giersch, Aaron R. Caldwell, Yoram Epstein, Nisha Charkoudian

**Affiliations:** ^1^Military Performance Division, U.S. Army Research Institute of Environmental Medicine, Natick, Massachusetts, United States; ^2^Thermal and Mountain Medicine Division, U.S. Army Research Institute of Environmental Medicine, Natick, Massachusetts, United States; ^3^School of Public Health, Faculty of Medicine, Tel Aviv University, Tel Aviv, Israel

**Keywords:** body size, body temperature, heat stress, military physiology, thermoregulation

## Abstract

Risk of exertional heat stroke (EHS) is an ongoing challenge for United States military personnel, for athletes and for individuals with occupational stressors that involve prolonged activity in hot environments. Higher body mass index (BMI) is significantly associated with increased risk for EHS in activity duty U.S. Soldiers. During exercise, heat is generated primarily by contracting skeletal muscle (and other metabolically active body mass) and dissipated based on body surface area (BSA). Thus, in compensable environments, a higher BSA·mass^−1^ may be a benefit to heat dissipation and decrease the risk of EHS. The purpose of the present analysis was to test the hypothesis that BSA·mass^−1^ ratio is an important biophysical characteristic contributing to the risk of EHS. We employed a matched case-control approach, where each individual with a diagnosis of EHS was matched to five controls who were never diagnosed with EHS but were in the same unit and had the same job title. We used a multivariate conditional logistic regression model including variables of BSA·mass^−1^, sex, age, military rank, and race. BSA·mass^−1^ significantly predicted EHS risk (*P* = 0.006), such that people with higher BSA·mass^−1^ were at lower risk of developing EHS when controlling for other potential factors such as age and race. This relationship persisted after adjustment for other anthropometric measures of body size including weight, BMI, and BSA. These data suggest that biophysical factors play an important role in EHS risk, particularly in a healthy military-aged cohort of men and women.

**NEW & NOTEWORTHY** With the impacts of climate change yielding higher average ambient temperatures over time, the incidence of EHS for individuals participating in outdoor activities may consequently increase. With the larger sample size in this study compared with prior research in this field, we were able to use various methods that had not been applied before. For example, we were able to mutually adjust for different measurements of body size to understand which metric had the highest association with EHS risk. Understanding factors that may be modifiable may be important for developing interventions to counteract the increased risk of EHS associated with climate change.

## Graphical abstract

**Figure:**
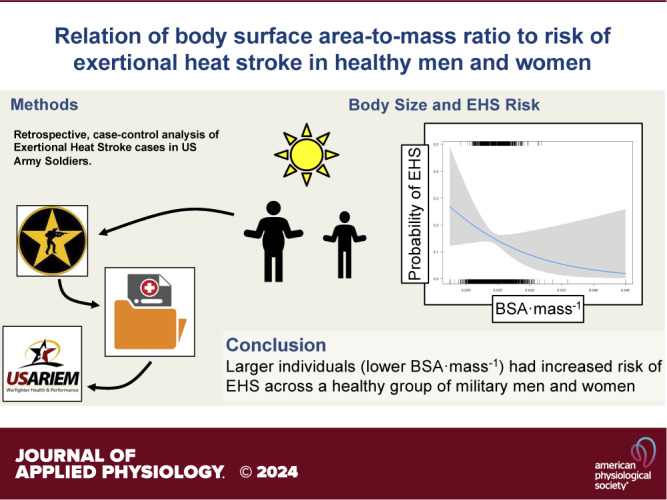

## INTRODUCTION

Exertional heat stroke (EHS) is an ongoing threat for those who are physically active, including athletes, occupational workers, and military personnel. In 2021, there were 488 cases of EHS in the U.S. Military (1), which is in line with the approximately 500 cases on average in previous years (1, 2). From 2011–2020, global surface temperatures were on average 1–2°C higher than 1850–1900 temperatures. The rate of increase in temperature has been faster in the last 50 years compared with any other 50-year period over the last 2,000 years (3). With the impacts of climate change yielding higher average ambient temperatures over time, the incidence of EHS for individuals participating in outdoor activities will likely consequently increase (4). Although some interventions can minimize heat illness cases, certain training, deployment, and combat scenarios that place military personnel at higher risk for EHS are unavoidable (5).

We previously showed a significant effect of body mass index (BMI) on EHS risk at a population level (2). Although BMI is a metric often used by health professionals and the U.S. Military to quantify body size, it is not particularly physiologically or biophysically informative in terms of thermoregulation. Individuals with higher BMI are often larger in stature, which decreases their body surface area-to-mass ratio (BSA·mass^−1^). Body surface area (BSA) is a primary factor for determining heat loss as humans dissipate heat from the skin surface via sweating in combination with increased skin blood flow (6). Body mass, particularly muscle mass, is a primary factor in heat production (7, 8) For a given absolute workload, individuals with lower BSA·mass^−1^ have been postulated to be at a “disadvantage” in terms of thermoregulation because of the relative decrease in capacity for heat dissipation relative to heat generation (9).

BMI has increased in military servicemembers, in line with the known general population, over the past decades (10–13). The combination of increasing average BMI, body size, and global temperature could have additive or synergistic effects to increase the risk of EHS. There is a critical need for the military to understand the impact of biophysical properties of body size, as they relate to EHS risk, to ensure appropriate guidance is being developed to reduce heat illness incidence. The primary aim of the present investigation, therefore, was to assess how biophysical factors related to body size impact EHS risk in a primarily young, active military population. Specifically, we aimed to evaluate which body size metrics (i.e., BMI, BSA, BSA·mass^−1^, or mass) contribute the most to a higher risk of EHS.

Our previous work indicated an effect of age on EHS risk, where younger individuals had an increased risk compared with their older counterparts (within an overall young, healthy military cohort) (2). This contrasts with previous findings showing that older individuals experience greater thermoregulatory strain due to blunted thermoeffector responses (14). A secondary aim of the present work was to test whether this difference was unique to our military population and was related to lower-ranking individuals (who may perform more high-risk activities) often being younger than their higher-ranking counterparts.

Thus, in the present analysis, we tested the hypotheses *1*) that BSA·mass^−1^ ratio, as a continuous variable, is negatively associated with risk for EHS in healthy, primarily young (90% < 36 yr old) military service members, and *2*) that military rank is negatively associated with risk in this population.

## MATERIALS AND METHODS

### Population

We conducted a matched nested case-control study within a cohort consisting of all U.S. Army Soldiers who were enlisted or commissioned from Jan 1, 2016, to December 31, 2021. Data were extracted from the Soldier Performance Health and Readiness Database (SPHERE) a large data repository that incorporates all medical, administrative, and physical records for the entire Army maintained at the U.S. Army Research Institute of Environmental Medicine (USARIEM). This study was approved by the Institutional Review Board at the U.S. Army Research Institute of Environmental Medicine, which approved a HIPAA waiver of authorization for individual written consent and complies with the World Medical Association Declaration of Helsinki—Ethical Principles for Medical Research Involving Human Subjects.

### Case Definition

Incident cases of EHS were identified using International Classification of Disease codes version 10 (ICD-10) found in the military health system data repository (MDR). The MDR is a comprehensive database that captures all medical encounter data where a soldier was treated in a military treatment facility or where TRICARE insurance was used. An incident case of EHS was defined as any soldier who received a diagnosis from the following list of ICD-10 codes: T67.02XA, T67.02XD, and T67.02XS. The date of the incident event was designated as the first time one of the ICD-10 codes appeared in a case’s medical record.

### Control Definition

We conducted risk set matching, where every case was matched to five controls who had never received a diagnosis of EHS (15). Each control was matched to a case based on the calendar day of the EHS diagnosis, unit ID, and military occupational specialty. This matching allows for analysis of individual risk factors while minimizing physical activity or environmental influence, given that controls would have been at the same location on the same day within the same unit and with the same job expectations doing similar activities at the same time as the EHS cases.

### Anthropometric Measurements and Body Size Definitions

We used the closest height and weight measurements to the EHS event for the cases and to the match date for the controls. Height and weight were captured from soldiers’ bi-annual body composition assessment. Height and weight were measured following a standardized procedure outlined in Army Regulation (AR) 600-9. Briefly, AR 600-9 requires height and weight measurements in socks, wearing standardized army-issued lightweight shirts, shorts and, for women, sports bras. Those who did not have height and weight measurements within 180 days of the index date were excluded from further analysis. BMI was calculated from the height (m) and weight (kg) using the standard formula (kg/m^2^) (16). BSA was calculated using the formula defined by Dubois and Dubois BSA (m^2^) = kg^0.425^ × m^0.725^ × 71.84) and this was used to calculate the BSA to mass(kg) ratio (BSA·mass^−1^) (17).

### Analysis

We used individual conditional logistic regression models to produce standardized betas to individually evaluate the relationship between weight, BMI, BSA, and BSA·mass^−1^ to EHS. We further evaluated and compared the strength of the contribution of weight, BMI, BSA, and BSA·mass^−1^ to EHS risk by mutually adjusting all covariates in the same model and extracting the standardized betas to compare the strength of the association while accounting for the fact that the covariates are related to each other. All models were adjusted for sex, age, race, time in service, and time since the last height and weight measurement.

## RESULTS

Within the study population, EHS cases on average tended to be of younger age, lower rank, have slightly higher BMI, and have a shorter time from their last height and weight measurement than the non-EHS controls. In terms of overall average values, BSA and BSA·mass^−1^ appeared to be similar between groups (Table 1). The range of ages for this cohort was 17–58 yr; 90% of the population were below 36 yr old and 75% were younger than 29 yr old.

**Table 1.** Population characteristics by EHS status

	EHS (*n* = 744)	No EHS (*n* = 3,720)
	Means ± SD or *n* (%)	Means ± SD or *n* (%)
BSA, m^2^	1.99 ± 0.18	1.99 ± 0.18
BSA·mass^−1^, m^2^/kg	0.0244 ± 0.002	0.0243 ± 0.005
Body mass, kg	82.53 ± 12.46	82.48 ± 13.20
BMI, kg/m^2^	26.51 ± 3.10	26.44 ± 3.55
Age, yr	24.57 ± 5.52	26.08 ± 6.80
Time since last weight/height measurement, days	78.08 ± 61.91	86.05 ± 57.57
Sex		
Male	683 (91.8)	3388 (91.1)
Female	61 (8.2)	332 (8.9)
Rank		
E1–E3	329 (44.2)	1344 (36.2)
E4–E6	258 (34.7)	1462 (39.3)
E7–E9	24 (3.2)	253 (6.8)
O1–O3	121 (16.3)	569 (15.3)
O4–O6	12 (1.6)	90 (2.4)

SD, standard deviation; *n*, number, EHS, exertional heat stroke; BSA, body surface area; BSA·mass^−1^, body surface area-to-mass ratio; E, enlisted soldiers at the following ranks: E1–E3 = Private to Private First Class; E4–E6 = Corporal to Staff Sergeant; E7–E9 = Sergeant First class to Command Sergeant Major; O = Officers at the following ranks: O1–O3 = Second Lieutenant to Captain, O4–O6 = Major to Colonel.

**Figure 1.**
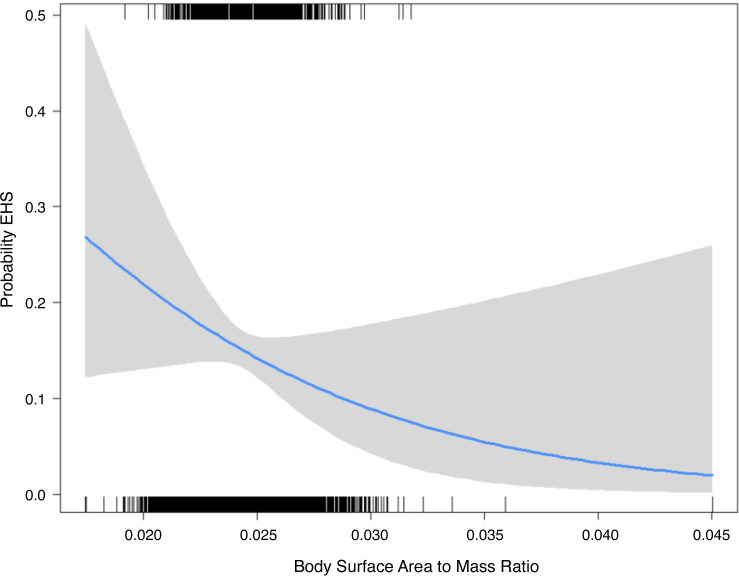
The probability of having an EHS by BSA·mass^−1^. The gray area represents the 95% confidence intervals for the probability (blue line) at each BSA·mass^−1^ amount. The rug plot at the *top* of the figure represents the BSA·mass^−1^ distribution for the cases, and the rug plot at the *bottom* of the figure represents the BSA·mass^−1^ distribution for the controls. BSA, body surface area; EHS, exertional heat stroke.

Within the individual adjusted models, BSA (*P* = 0.09) was not associated with EHS while increasing body weight (*P* = 0.017), increasing BMI (*P* = 0.005), and decreasing BSA·mass^−1^ (*P* = 0.006) were significantly associated with EHS. Figure 1 demonstrates the probability of developing EHS as BSA·mass^−1^ increases. When models were further adjusted for body weight, BSA·mass^−1^ (*P* = 0.021) maintained its significant inverse associations with EHS while BMI (*P* = 0.118) was no longer significantly harmful and BSA (*P* = 0.119) remained nonsignificant. This suggests that the biophysical properties of body size (BSA·mass^−1^), not simply body size are the driving factor for increasing risk of EHS. Within the models that are mutually adjusted for BSA, BSA·mass^−1^, BMI, and weight, all covariates became significant (Table 2). The standardized betas both in the individual models and the mutually adjusted models demonstrated the strongest predictor of EHS is BSA·mass^−1^.

**Table 2.** Nonmutually adjusted models and a mutually adjusted model with standardized betas to assess strength of significant relationship between body size variables and EHS risk

	Nonmutually Adjusted Models					Mutually Adjusted Models				
Variable	Odds Ratio	95% CI		*P* Value	Standardized β	Odds Ratio	95% CI		*P* Value	Standardized β
BSA	1.000	1.000	1.001	0.093	0.044	0.984	0.973	0.996	0.006	−1.5796
BSA·mass^−1^	0.931	0.888	0.975	0.003	−0.148	0.431	0.259	0.719	0.001	−2.0261
BMI	1.035	1.011	1.061	0.067	0.072	0.516	0.330	0.808	0.003	−1.2693
Weight	1.008	1.001	1.015	0.02	−0.060	1.275	1.056	1.539	0.001	1.7526

Model also adjusted for sex, age, race, time in service, and time since last height and weight measurement. An odds ratio greater than 1 indicates an increase in the odds of EHS associated with a one unit change in the model variable. An odds ratio less than 1 indicates a reduction in the odds of EHS associated with a one unit change in the model variable. BSA, body surface area (unit = 0.01 m^2^); BSA·mass^−1^, body surface area-to-mass ratio (unit = 0.01 m^2^/kg); BMI, body mass index, EHS, exertional heat stroke.

**Figure 2.**
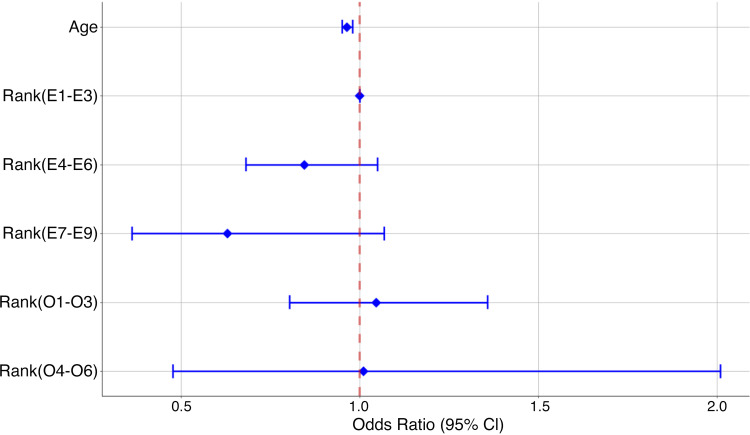
Odds ratios and 95% confidence intervals from the adjusted models demonstrating the relationship between EHS and age and rank. Models were adjusted for BSA·mass^−1^, sex, race, age, and time since last height and weight measurement. BSA, body surface area; EHS, exertional heat stroke.

Within the models, age maintained a significant relationship with EHS even after adjustment for rank (Fig. 2). This may indicate that decreasing age may play a role increased risk of EHS.

## DISCUSSION

In the present analysis, we have demonstrated that lower BSA·mass^−1^ was associated with increased risk of EHS across a healthy group of men and women ranging in age from 17 yr to 58 yr (90% of whom were below the age of 36). Interestingly from a military perspective, higher rank among enlisted service members was not associated with EHS compared with the lowest ranks (E1–E3), while younger age persisted as a risk factor for increased risk of EHS. Our model results further suggest that the primary driver of increased risk associated with EHS is lower BSA·mass^−1^. Specifically, BSA·mass^−1^ appears to be driving the increased risk previously observed with BMI (2).

Current trends indicate that body weight and BMI are increasing at a population level, and that the rates of obesity across the United States and other developed countries are at all-time high levels. In general, increased body size, including higher BMI and/or body weight, are associated with lower BSA·mass^−1^, because surface area increases at a relatively lower rate than mass as body size increases.

Although there is some debate on this issue (see discussion below), the idea of an advantage for higher BSA·mass^−1^ is that heat is generated based on body mass (e.g., exercising muscle mass) and dissipated based on surface area (e.g., skin blood flow and sweating in humans). Thus, in a compensable environment (an environment where it is possible to dissipate heat by the usual thermoregulatory mechanisms), a smaller person is at an advantage for thermoregulatory heat dissipation. An important caveat is that the environment must be compensable. For example, if ambient conditions are very hot and humid, or if the person is completely encapsulated (such as with military or industrial workers in protective clothing), this is considered an “uncompensable” heat stress. In such conditions, the individual would gain heat from their environment or dissipate less heat from the body having a higher BSA·mass^−1^ would not be an advantage. However, in most conditions experienced by soldiers in training, the conditions would fall into the “compensable” category since extremes of heat and humidity associated with uncompensable environments would (by regulation) be associated with canceled or modified training events (18).

For the military and athletic populations, it is important to note that the national and international trends for increasing BMI (and lower BSA·mass^−1^) persist even within an active population like the U.S. Army. This trend for people to get bigger therefore compounds the fact that rising average temperatures and increased extreme weather events across the globe are already creating increased risk for exertional heat illness. In terms of public health considerations relative to climate change, this appears to be creating a “perfect storm” for individuals such as soldiers who need to maintain activity in hotter environments.

Our present results provide a new perspective on an interesting and complex discussion in the thermoregulatory literature that has been going on for several decades: what is the role of body size and shape in thermoregulatory responses to exercise in hot environments? On one hand, some studies suggest small body size is an advantage (9); whereas others suggest that larger individuals have smaller increases in core temperature for a given exercise-heat stress scenario (19, 20).

Our conclusion that high BSA·mass^−1^ is an advantage relative to risk for heat stroke may seem to be at odds with the previous conclusion that large body size/higher body mass was beneficial in preventing hyperthermia because of the role of the larger mass as a “heat sink” during metabolic heat production, and therefore lower BSA·mass^−1^ was associated with slower increases in core temperature during periods of continuous exercise for 30–60 min (19, 20). The ability of body mass itself to act as a heat sink is most clearly seen during non-weight-bearing exercise (19), whereas during weight-bearing exercise, it is more likely that body weight itself contributes to the exercise load and therefore to metabolic heat production (20). In the present analysis, it is likely that most, if not all, of the activities performed were weight bearing, in some cases, including heavy load carriage (e.g., ruck marches). Furthermore, in situations where there are repeated work/rest cycles over several hours or more, people with larger body mass (and more thermal “inertia”) would have smaller decreases in body temperature (for a given rest period[s]), resulting in potentially greater total cumulative increases in temperature over time.

It is also important to note that we did not specifically evaluate the rate of increase in core temperature in this study. Although the rate of increase of core temperature (Tc) is a major contributor to the development of EHS, it is not the only contributing factor (21, 22); cardiovascular strain is also a major contributor (23). Absolute levels of skin blood flow contribute to cardiovascular strain associated with work in the heat (24–26), and larger people (with larger total BSA and smaller BSA·mass^−1^) would have a greater absolute requirement for skin blood flow for a similar absolute exercise intensity. Certainly, more work is needed to clarify the mechanisms linking body size and EHS risk.

A strength of the present study was that we used population-level data from a large and diverse sample of soldiers to evaluate the relationship between body size and EHS. We were able to match controls with EHS cases on unit location and job title on the day of the EHS event, which likely minimized confounding by differences in activities and temperature. In this context, a limitation was that the EHS cases included in this analysis include individuals who almost certainly developed EHS during ruck marches and timed runs (22). As discussed above, carrying a weighted rucksack in the Army Combat Uniform (ACU) would likely drastically reduce any benefit to having greater BSA·mass^−1^. Unfortunately, in this data, we are unable to determine which activities and clothing conditions of the EHS cases are included in the analysis as it is not information present in the medical records we used. However, this likely means that the influence of BSA·mass^−1^ is even stronger than what we have identified here, which we might have seen more clearly if we had been able to remove the potential confounds of the present data in terms of uniform, load carriage, and related issues.

With regard to the persistent influence of (younger) age in our cohort, we recognize that this is different from population-based analyses regarding the influence of age on heat illness risk (27). However, as noted in our previous work, we propose that behavioral/strategic differences across age groups might contribute to the increased risk of heat illness seen in younger soldiers. For example, younger groups might be more likely to have so-called “excessive” motivation, which would prevent them from making the best decisions to stop or take breaks when they were receiving extreme discomfort signals from thermal perception afferent pathways (28). The external motivation as a risk factor is also particularly relevant to this military cohort as there are times when performance on physical tasks is related to promotion potential (22), which we believe contributes to the higher risk in soldiers of younger age in this analysis. Furthermore, we cannot rule out the influence of survivorship bias. Within our cohort, the older soldiers may have other yet unmeasured characteristics that are protective against EHS while other individuals of a similar age, who have increased EHS risk, have already left military service. In essence, older individuals who are at higher risk of EHS may have been disqualified from military service for other reasons (e.g., low cardiorespiratory fitness).

In terms of interpretation of the present data, it is important to note that our data specifically relate to EHS, not passive (or “classic”) heat stroke, which is more commonly seen in older individuals and is usually not associated with exercise. It may be worthwhile for public health scientists to investigate how body size, BMI, or BSA·mass^−1^ impacts classic heat stroke risk, as BMI tends to increase with age along with other factors that contribute to heat stroke risk.

In summary, we have identified important quantitative influences of body size, as quantified in terms of body surface area to mass ratio, on the risk of exertional heat stroke risk in a large group of primarily young, healthy men and women who are active-duty soldiers in the U.S. Army. Based on general demographic information, this group appears to be representative of individuals in this age range in general in the United States. This influence of BSA·mass^−1^, in combination with trends for increased body size (decreased BSA·mass^−1^) and increasing environmental temperatures due to climate change, represent important areas of focus for future work in terms of the public health impact of climate change across the world.

## DATA AVAILABILITY

The use of population level PHI/PII from Department of Defense data agencies precludes the data from being made available due to third party restrictions outlined in data use agreements.

## GRANTS

This project was supported by funding from Military Operational Medicine Research Program, US Army Medical Research and Development Command (MO230002).

## DISCLAIMERS

The views, opinions, and/or findings contained in this article are those of the authors and should not be construed as an official United States Department of the Army position, or decision, unless so designated by other official documentation. Approved for public release, distribution unlimited. Citations of commercial organizations and trade names in this report do not constitute an official Department of the Army endorsement or approval of the products or services of these organizations.

## DISCLOSURES

No conflicts of interest, financial or otherwise, are declared by the authors.

## AUTHOR CONTRIBUTIONS

K.M.T., G.E.W.G., and A.R.C. conceived and designed research; K.M.T. analyzed data; K.M.T., G.E.W.G., A.R.C., Y.E., and N.C. interpreted results of experiments; K.M.T. prepared figures; K.M.T., G.E.W.G., and N.C. drafted manuscript; K.M.T., G.E.W.G., A.R.C., Y.E., and N.C. edited and revised manuscript; K.M.T., G.E.W.G., A.R.C., Y.E., and N.C. approved final version of manuscript.
